# Reduced *GEN1* Expression Is Associated with Elevated DNA Damage and Impaired Proliferation in Endometriosis-Derived Endometrial Organoids

**DOI:** 10.3390/ijms27073034

**Published:** 2026-03-26

**Authors:** Berivan Guzelbag, Nazli Ece Gungor, Hadice Karahan, Alireza Maghsoudi, Engin Oral

**Affiliations:** 1 Department of Obstetrics and Gynecology, Haseki Training and Research Hospital, Health Sciences University, 34270 Istanbul, Türkiye; drguzelbag@gmail.com; 2 Department of Molecular and Medical Genetics, Institute of Graduate Studies, Biruni University, 34020 Istanbul, Türkiye; 3 Histology and Embryology Department, Medical School, Biruni University, 34020 Istanbul, Türkiye; ngungor@biruni.edu.tr; 4 Molecular Biology and Genetics Department, Faculty of Natural Sciences and Engineering, Biruni University, 34020 Istanbul, Türkiye; hadicekarahan00@gmail.com; 5 Department of Stem Cell and Tissue Engineering, Graduate School of Health Sciences, Istinye University, 34010 Istanbul, Türkiye; alirezamaqsudi411@gmail.com; 6 Department of Obstetrics and Gynecology, Norrland University Hospital, Umeå University, 90187 Umea, Sweden

**Keywords:** DNA repair, endometrial organoids, endometriosis, *GEN1*, homologous recombination, γH2AX

## Abstract

Endometriosis affects approximately 10% of reproductive-age women and is associated with genomic instability; however, the contribution of specific DNA repair deficiencies remains poorly understood. This study investigated the expression and function of *GEN1*, a Holliday junction resolvase critical for homologous recombination, in patient-derived endometrial epithelial organoids (EEOs). Endometrial tissue was obtained by pipelle biopsy from women with laparoscopically confirmed endometriosis (*n* = 3, stage III–IV) and controls without endometriosis (*n* = 3). *GEN1* mRNA and protein expression were reduced in primary endometrial cells from endometriosis patients compared with controls (mRNA: 0.52 ± 0.14 vs. 1.00 ± 0.19, *p* = 0.05; immunofluorescence intensity: 0.54 ± 0.18 vs. 1.00 ± 0.22, *p* = 0.05). Patient-derived EEOs from the endometriosis group showed trends toward lower formation efficiency (18.4 ± 5.6% vs. 25.2 ± 6.8%, *p* = 0.10) and reduced mean diameter (124.6 ± 34.2 vs. 155.8 ± 32.6 µm, *p* = 0.10). RNA interference (RNAi)-mediated *GEN1* knockdown reduced proliferation in both groups, with a more pronounced effect in endometriosis-derived EEOs (49.7% vs. 39.5% reduction, *p* = 0.05). Endometriosis-derived EEOs exhibited elevated baseline γH2AX (phosphorylated histone H2AX) immunofluorescence compared with controls (2.32 ± 0.44 vs. 1.00 ± 0.28, *p* = 0.05), indicating increased DNA double-strand break accumulation. Furthermore, *GEN1* knockdown directly increased γH2AX intensity in both groups, with endometriosis-derived EEOs showing a greater absolute increase (Δ1.26 vs. Δ0.72). To our knowledge, this study provides the first organoid-based evidence that *GEN1* is downregulated in endometriosis and functionally linked to impaired proliferation and elevated DNA damage, suggesting a potential contribution of homologous recombination dysregulation to endometriosis pathogenesis.

## 1. Introduction

Endometriosis is a chronic, oestrogen-dependent inflammatory disease characterised by the presence of endometrial-like tissue outside the uterine cavity, affecting approximately 10% of women of reproductive age worldwide, corresponding to an estimated 190 million individuals globally [1,2]. Clinically, the disease manifests as chronic pelvic pain, dysmenorrhoea, dyspareunia, and infertility, with a diagnostic delay averaging 7–10 years due to symptom normalisation and the lack of non-invasive diagnostic tools [1]. According to the most widely accepted retrograde menstruation theory, menstrual debris implants onto peritoneal surfaces; however, although retrograde menstruation occurs in the majority of women, endometriosis develops in only a subset [3,4]. This observation underscores the multifactorial nature of the disease, emphasising the complex interplay between genetic predisposition, epigenetic modifications, immune dysfunction, and environmental influences [3]. While genome-wide association studies have identified numerous genetic loci associated with endometriosis, these variants explain only a small proportion of disease heritability [5,6], and functional studies are needed to clarify the specific molecular pathways contributing to disease development.

Oxidative stress plays a central role in the pathogenesis of endometriosis [7,8]. Iron released from erythrocytes reaching the pelvic cavity during retrograde menstruation leads to excessive production of reactive oxygen species (ROS) through the Fenton reaction [8]. This oxidative environment contributes to genomic instability by causing lipid peroxidation, protein oxidation, and DNA damage [7,8]. The accumulation of DNA double-strand breaks and base damage in endometriotic cells has been associated with defective DNA repair mechanisms [9,10]. The homologous recombination (HR) pathway is critical for the error-free repair of DNA double-strand breaks, and variants in genes involved in this pathway threaten genome integrity [11]. Polymorphisms in DNA repair genes such as *XRCC1*, *XRCC3*, and *BLHX* have been reported to be associated with endometriosis risk and chromosome instability [12,13]. These findings suggest that deficiencies in DNA repair mechanisms play an important role in the molecular pathogenesis of endometriosis [9,10,14].

*GEN1* (Gap Endonuclease 1) is a Holliday junction resolvase enzyme that plays a critical role in homologous recombination [15]. Resolution of four-way Holliday junction structures formed during the repair of DNA double-strand breaks is essential for maintaining genome integrity and chromosome segregation [15]. *GEN1* is a member of the Rad2/XPG nuclease family and specifically binds to Holliday junctions, resolving them by introducing symmetric nicks in the non-crossing strands [11,16]. Unlike other Rad2/XPG nucleases, human *GEN1* protein contains a chromodomain for DNA recognition and binding [16]. Nuclear exclusion and activation of *GEN1* are regulated throughout the cell cycle, and this spatial control is critical for preventing genomic instability by restricting *GEN1* activity to mitosis [15,17]. Furthermore, *GEN1* has been shown to play a role in maintaining centrosome integrity, and *GEN1* deficiency has been associated with aberrant centrosome numbers, multiple mitotic spindle poles, and spontaneous DNA damage [18]. A recent whole-exome sequencing study identified a *GEN1* gene variant (c.1574C>T, p.Ser525Leu) associated with endometriosis in a Turkish family, highlighting the potential role of DNA repair mechanisms in endometriosis pathogenesis [19].

Three-dimensional organoid culture systems have become powerful tools for disease modelling by recapitulating the physiological features of human tissues in vitro [20,21]. Human endometrial organoids were first successfully established by two independent groups in 2017, demonstrating long-term expansion capacity, genetic stability, and hormonal responsiveness [22,23]. Endometrial organoids preserve the cellular heterogeneity, transcriptomic profile, and functional characteristics of the original tissue, providing a unique platform for investigating endometrial physiology and pathology [23]. In endometriosis research, patient-derived organoids have been shown to capture clinical heterogeneity of the disease, reflect molecular features of ectopic lesions, and be suitable for drug screening studies [24]. These organoid models maintain disease-specific characteristics including aberrant activation in integrin, PI3K-AKT, and WNT signalling pathways, enabling identification of potential therapeutic targets [24]. The recent development of internationally harmonised protocols for endometrial organoid culture further supports the standardisation and reproducibility of this model system [25,26]. Furthermore, the application of functional genomic approaches, including gene silencing and gene editing technologies, to patient-derived organoids offers new opportunities for investigating the biological consequences of specific genetic variants implicated in endometriosis [25].

Although the critical role of the *GEN1* gene in DNA repair mechanisms and its potential association with endometriosis have been suggested, including the identification of a missense variant (c.1574C>T, p.Ser525Leu) in a Turkish family [19], the functional consequences of *GEN1* deficiency in endometrial cells remain unexplored. Patient-derived endometrial organoids provide an ideal platform for investigating the functional effects of gene deficiencies at the cellular level [24]. In this study, we aimed to establish and characterise endometrial organoids derived from endometriosis patients and controls, compare *GEN1* expression between groups, and evaluate the effects of *GEN1* deficiency on cell proliferation and DNA damage accumulation. Our study represents the first organoid-based investigation exploring the functional role of *GEN1* in endometriosis pathogenesis, and the findings may contribute to understanding the molecular basis of the disease and elucidating the role of DNA repair dysregulation in endometriosis pathogenesis.

## 2. Results

### 2.1. Study Population

A total of six women were enrolled in this study: three patients with laparoscopically and histopathologically confirmed endometriosis and three control subjects without laparoscopic evidence of endometriosis. The demographic and clinical characteristics of the participants are summarised in Table 1. No statistically significant differences were observed between groups for age, body mass index, menstrual cycle day, or parity (all *p* > 0.05, exact Mann–Whitney U test). All endometrial samples were collected during the proliferative phase of the menstrual cycle (cycle days 8–12).

### 2.2. GEN1 Is Expressed in Human Endometrial Epithelial and Stromal Cells

**Figure 1:**
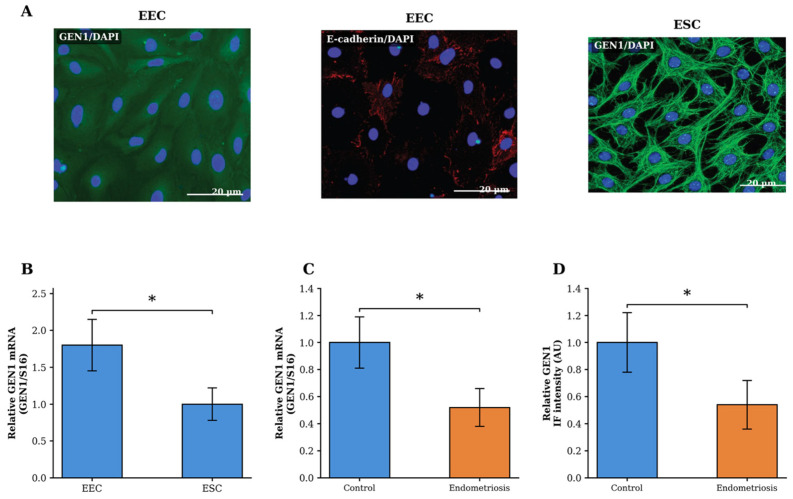
*GEN1* expression in primary endometrial cells. (**A**) Immunofluorescence staining of endometrial epithelial cells (EEC; left and middle) and endometrial stromal cells (ESC; right). *GEN1* (green) exhibited prominent nuclear localisation in both cell types. E-cadherin (red) confirmed epithelial identity of EEC. Nuclei were counterstained with DAPI (blue). Scale bars: 20 µm. (**B**) Relative *GEN1* mRNA expression in EEC and ESC, normalised to S16. (**C**) Relative *GEN1* mRNA expression in control and endometriosis tissue samples, normalised to S16. (**D**) Quantification of *GEN1* immunofluorescence intensity in control and endometriosis samples. Data are presented as mean ± SD (*n* = 3 per group). * *p* = 0.05, Mann–Whitney U test.

To investigate *GEN1* expression in human endometrial tissue, immunofluorescence analysis was performed on freshly isolated primary endometrial epithelial cells (EECs) and endometrial stromal cells (ESCs) from control subjects (*n* = 3). Confocal microscopy revealed that *GEN1* protein was expressed in both cell types, with prominent nuclear localisation consistent with its known function as a Holliday junction resolvase (Figure 1A). E-cadherin staining in epithelial cells confirmed the preservation of cell–cell junctions and epithelial architecture during the isolation procedure (Figure 1A). RT-qPCR analysis demonstrated that *GEN1* mRNA was expressed in both EECs and ESCs, with approximately 1.8-fold higher expression levels detected in epithelial cells compared with stromal cells (Figure 1B).

### 2.3. GEN1 Expression Is Reduced in Endometriosis Patients

*GEN1* mRNA and protein levels were compared in primary endometrial cells obtained from patients with endometriosis (*n* = 3) and control subjects (*n* = 3). RT-qPCR analysis revealed that *GEN1* mRNA expression was reduced in the endometriosis group compared with controls (0.52 ± 0.14 vs. 1.00 ± 0.19, *p* = 0.05) (Figure 1C). Immunofluorescence intensity analysis demonstrated a similar reduction in *GEN1* protein levels in endometriosis samples compared with the control group (0.54 ± 0.18 vs. 1.00 ± 0.22, *p* = 0.05) (Figure 1D). This reduction was observed in both epithelial and stromal cell populations.

### 2.4. Establishment and Characterisation of Endometrial Organoids

**Figure 2:**
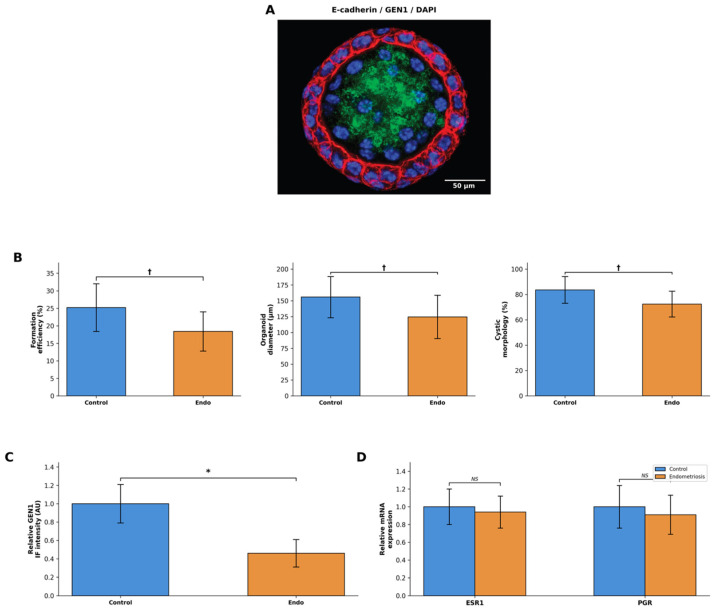
Characterisation of endometrial organoids. (**A**) Representative confocal image of a control EEO immunostained for E-cadherin (red), *GEN1* (green) and DAPI (blue), demonstrating epithelial organisation with luminal architecture. Scale bar: 50 µm. (**B**) Organoid formation efficiency (%), mean organoid diameter (µm) and proportion of cystic morphology (%) in control and endometriosis groups. (**C**) Quantification of *GEN1* immunofluorescence intensity in control and endometriosis EEOs. (**D**) Relative mRNA expression of oestrogen receptor alpha (ESR1) and progesterone receptor (PGR) in control and endometriosis EEOs, normalised to S16. NS, not significant. Data are presented as mean ± SD (*n* = 3 per group). * *p* = 0.05; † *p* = 0.10 (trend), Mann–Whitney U test.

Isolated single cells and small epithelial fragments obtained from both control subjects and endometriosis patients were embedded in Matrigel and allowed to form EEOs over 7–10 days. The resulting EEOs recapitulated the epithelial organisation of the endometrium, exhibiting well-defined luminal structures and E-cadherin-positive cell–cell junctions (Figure 2A).

EEO formation efficiency was lower in the endometriosis group compared with controls (18.4 ± 5.6% vs. 25.2 ± 6.8%, *p* = 0.10), representing a trend toward reduced EEO-forming capacity (Figure 2B). The mean EEO diameter was also reduced in endometriosis-derived EEOs compared with control EEOs (124.6 ± 34.2 µm vs. 155.8 ± 32.6 µm, *p* = 0.10), and a lower proportion of endometriosis EEOs displayed cystic morphology with luminal structures (72.4 ± 10.2% vs. 83.6 ± 10.5%, *p* = 0.10) (Figure 2B).

Immunofluorescence analysis demonstrated that *GEN1* was expressed in the EEO epithelial compartments in both groups, with nuclear enrichment co-localising with E-cadherin-positive regions (Figure 2A). *GEN1* immunofluorescence intensity was reduced in endometriosis-derived EEOs compared with control EEOs (0.46 ± 0.15 vs. 1.00 ± 0.21 AU, *p* = 0.05) (Figure 2C).

To assess whether EEOs maintained endometrial epithelial characteristics, hormone receptor expression was evaluated. RT-qPCR analysis showed that ESR1 mRNA levels were comparable between groups (control: 1.00 ± 0.20 vs. endometriosis: 0.94 ± 0.18, *p* > 0.05), as were PGR mRNA levels (control: 1.00 ± 0.24 vs. endometriosis: 0.91 ± 0.22, *p* > 0.05), indicating preserved hormonal receptor status in EEOs from both groups (Figure 2D).

### 2.5. Efficient RNAi-Mediated Knockdown of GEN1 in Organoids

**Figure 3:**
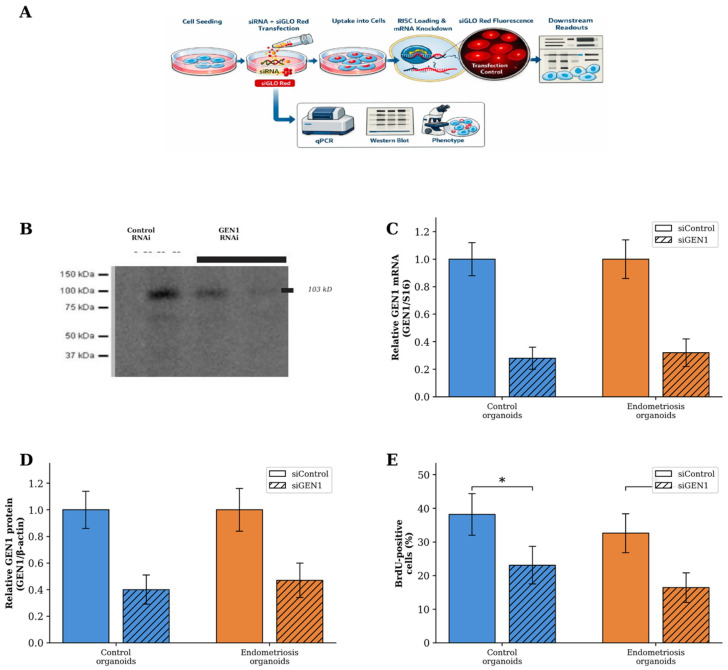
RNAi-mediated *GEN1* knockdown in endometrial EEOs and functional effects. (**A**) Schematic representation of the siRNA transfection workflow using siGLO Red as a transfection indicator. (**B**) Representative Western blot showing *GEN1* protein expression (103 kD) in control RNAi and *GEN1* RNAi-treated EEOs. (**C**) Relative *GEN1* mRNA expression following siRNA treatment in control and endometriosis EEOs, normalised to S16. Solid bars, siControl; hatched bars, si*GEN*. (**D**) Relative *GEN1* protein expression following siRNA treatment, determined by Western blot densitometry and normalised to β-actin. (**E**) BrdU incorporation assay showing the percentage of proliferating cells following *GEN1* knockdown in control and endometriosis EEOs. Data are presented as mean ± SD (*n* = 3 per group). * *p* = 0.05, Mann–Whitney U test. Knockdown efficiency (**C**,**D**) reported descriptively.

RNAi-mediated gene silencing was applied to organoids derived from both control subjects and endometriosis patients. Seven-day EEO cultures were transfected with *GEN1* -specific siRNA duplexes or non-targeting control siRNA at a final concentration of 20 nM (Figure 3A). Transfection efficiency, assessed by siGLO Red co-transfection, was 87.3 ± 5.4% in control EEOs and 85.8 ± 6.2% in endometriosis EEOs (*p* > 0.05).

RT-qPCR analysis confirmed efficient *GEN1* knockdown in both groups: 72 ± 8% reduction in control EEOs and 68 ± 10% reduction in endometriosis EEOs relative to respective non-targeting siRNA controls (Figure 3C). At the protein level, Western blot densitometry indicated a 60 ± 11% reduction in control EEOs and 53 ± 13% reduction in endometriosis EEOs (Figure 3D). Immunofluorescence imaging showed reduced *GEN1* signal in siRNA-treated EEOs from both groups, while EEO morphology was preserved following transfection.

### 2.6. GEN1 Knockdown Reduces Proliferation While Maintaining Epithelial Integrity

Cell proliferation and epithelial integrity were examined in siRNA-treated EEOs from both control and endometriosis groups. BrdU incorporation analysis revealed a reduction in the proportion of proliferating cells following *GEN1* knockdown in both groups. In control EEOs, the percentage of BrdU-positive cells decreased from 38.2 ± 6.2% to 23.1 ± 5.6% following *GEN1* siRNA treatment, corresponding to a 39.5% reduction (*p* = 0.05) (Figure 3E). Endometriosis-derived EEOs exhibited a more pronounced response to *GEN1* knockdown, with BrdU-positive cells decreasing from 32.6 ± 5.8% to 16.4 ± 4.4%, corresponding to a 49.7% reduction (*p* = 0.05) (Figure 3E).

E-cadherin immunofluorescence staining demonstrated that cell–cell junctions and epithelial polarisation were preserved in *GEN1* knockdown EEOs from both groups. No apparent difference in E-cadherin expression intensity or localisation pattern was observed between control siRNA and *GEN1* siRNA groups in either control or endometriosis-derived EEOs.

**Figure 4:**
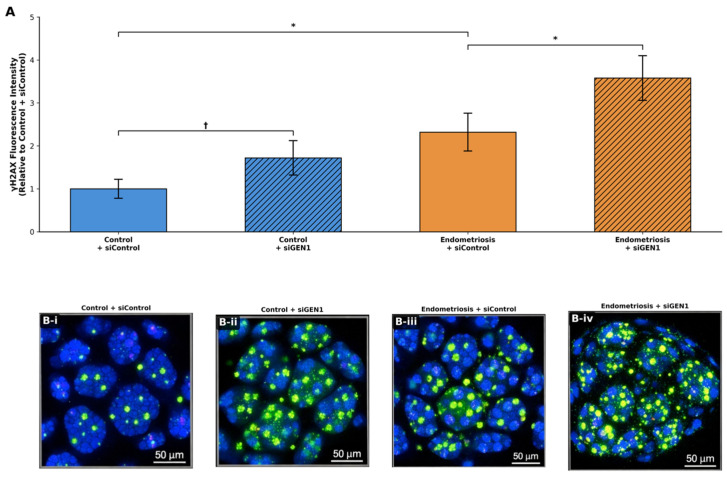
Effect of *GEN1* knockdown on DNA damage accumulation in EEOs. (**A**) Quantification of γH2AX immunofluorescence intensity in control and endometriosis EEOs following treatment with siControl or si*GEN1* at 48 h post-transfection. Values are expressed relative to control + siControl. (**B**) Representative confocal images of γH2AX (green) and DAPI (blue) staining in (**i**) control + siControl, (**ii**) control + si*GEN*, (**iii**) endometriosis + siControl, and (**iv**) endometriosis + si*GEN1* EEOs. Scale bars: 50 µm. Data are presented as mean ± SD (*n* = 3 per group). * *p* = 0.05; † *p* = 0.10 (trend), Mann–Whitney U test.

*GEN1* knockdown increased DNA damage in both groups. In control EEOs, γH2AX immunofluorescence intensity increased from 1.00 ± 0.22 to 1.72 ± 0.40 following si*GEN1* treatment (*p* = 0.10). Endometriosis-derived EEOs exhibited a more pronounced increase, from 2.32 ± 0.44 to 3.58 ± 0.52 (*p* = 0.05). In absolute terms, endometriosis-derived EEOs showed a greater increase in γH2AX intensity (Δ1.26) than control EEOs (Δ0.72), although the relative increase was lower (54% vs. 72%) owing to the already elevated baseline in the endometriosis group (Figure 4). Although the increase in γH2AX intensity in control EEOs did not reach statistical significance, the consistent directionality across groups supports a biological effect of *GEN1* depletion.

**Figure 5:**
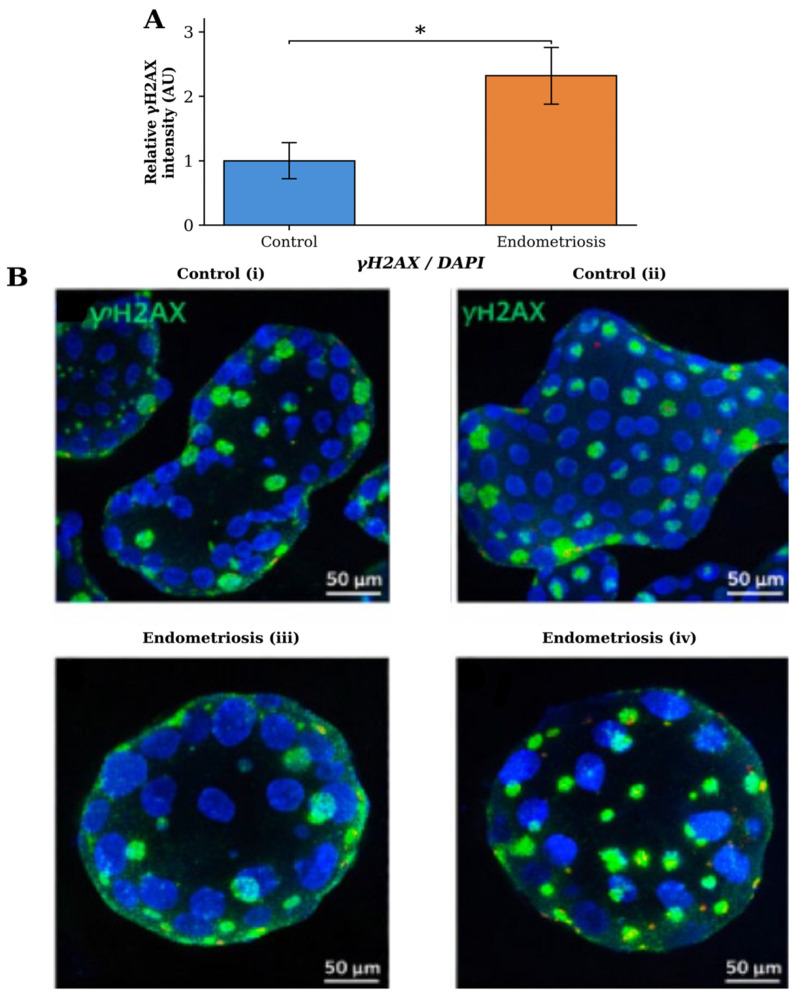
Baseline DNA damage in EEOs. (**A**) Quantification of γH2AX immunofluorescence intensity in control and endometriosis EEOs. (**B**) Representative confocal images of γH2AX (green) and DAPI (blue) staining in control (**i**,**ii**) and endometriosis (**iii**,**iv**) EEOs. Scale bars: 50 µm. Data are presented as mean ± SD (*n* = 3 per group). * *p* = 0.05, Mann–Whitney U test.

Endometriosis EEOs exhibited elevated baseline DNA damage compared with controls, as indicated by increased γH2AX immunofluorescence intensity (2.32 ± 0.44 vs. 1.00 ± 0.28, *p* = 0.05) (Figure 5).

## 3. Discussion

This study is the first to investigate the function of the DNA repair enzyme *GEN1* in endometrial EEOs and its potential role in endometriosis pathogenesis. Three key findings emerged: (1) *GEN1* mRNA and protein expressions were reduced by approximately half in eutopic endometrium of endometriosis patients compared with controls; (2) patient-derived EEOs maintained consistently low *GEN1* expression, and EEO formation efficiency showed a non-significant trend toward reduction in the endometriosis group (*p* = 0.10); and (3) RNAi-mediated *GEN1* knockdown reduced cell proliferation more pronouncedly in endometriosis-derived EEOs than in controls (49.7% vs. 39.5% reduction). These findings support the notion that defects in DNA repair mechanisms play an important role in endometriosis pathogenesis and provide the first organoid-based evidence for the potential functional significance of *GEN1* in this disease [8,19]. Furthermore, endometriosis-derived EEOs exhibited elevated baseline γH2AX immunofluorescence compared with controls, indicating increased DNA double-strand break accumulation in the disease state. This observation, combined with the heightened sensitivity to *GEN1* knockdown, supports the notion that endometriotic cells possess an underlying genomic fragility that may be further exacerbated by additional disruption of the homologous recombination pathway. Indeed, *GEN1* knockdown directly increased γH2AX immunofluorescence in both groups, with endometriosis-derived EEOs showing a greater absolute increase in γH2AX intensity (Δ1.26 vs. Δ0.72), consistent with a role for *GEN1* deficiency in exacerbating DNA damage accumulation.

The reduction in *GEN1* expression observed in the present study is consistent with previous studies demonstrating altered DNA repair gene expression in eutopic endometrium of women with endometriosis. Govatati et al. demonstrated significantly decreased *PTEN* protein expression in eutopic endometrium of endometriosis patients [27]. A transcriptome meta-analysis by Poli-Neto et al. revealed downregulation of genes related to DNA repair and cell cycle control in eutopic endometrium of women with endometriosis [28]. The *GEN1* expression reduction (~48%) observed in our study aligns with this general trend and suggests that the homologous recombination pathway may be compromised in endometriosis [10]. Notably, the association of a *GEN1* genetic variant with endometriosis risk reported by Kina et al. further supports the potential importance of *GEN1* in this disease [19]. Broader genetic evidence from GWAS studies has identified multiple loci associated with endometriosis susceptibility, though the specific contribution of DNA repair gene variants remains incompletely characterised [5,6]. Polymorphisms in DNA repair genes, including *XRCC1*, *XRCC3*, and *BLHX*, have been associated with endometriosis risk and chromosomal instability in lymphocytes from affected women [12,13]. Collectively, these genetic findings converge with our functional data to suggest that impaired homologous recombination, exemplified here by reduced *GEN1* expression, may represent one of the molecular mechanisms underlying the genomic instability observed in endometriosis.

The patient-derived EEO model employed in our study offers significant advantages over traditional two-dimensional cell culture and animal models for investigating endometriosis pathogenesis. EEOs allow long-term expansion while preserving the genetic and phenotypic characteristics of the original tissue and reflecting disease diversity [21,24]. As demonstrated by Boretto et al., endometriosis-derived EEOs exhibit disease-specific features and can recapitulate the original lesion following in vivo transplantation [24]. Gu et al. emphasised that EEOs demonstrate high stability and patient specificity compared with cell lines and animal models [29]. The non-significant trends toward lower EEO formation efficiency (18.4% vs. 25.2%, *p* = 0.10) and smaller EEO size (124.6 vs. 155.8 µm, *p* = 0.10) observed in our study are consistent with disease-specific morphological differences, though these should be interpreted cautiously given the small sample size. Similar morphological differences have been reported by Boretto et al. [22], demonstrating that ectopic EEOs grow more slowly and exhibit different morphology compared with those derived from healthy tissues [23].

The greater proliferation reduction in endometriosis EEOs following *GEN1* knockdown (49.7% vs. 39.5%, *p* = 0.05) is consistent with synthetic lethality, where pre-existing DNA repair defects sensitise cells to further pathway inhibition [30,31]. The already reduced basal *GEN1* expression in endometriosis EEOs may fall below a critical threshold upon RNAi, severely compromising homologous recombination capacity.

Iron released during retrograde menstruation leads to excessive production of reactive oxygen species through the Fenton reaction, causing DNA damage [7,8]. The landmark study by Anglesio et al. detected somatic mutations in 79% of deep infiltrating endometriosis lesions and identified mutations in known cancer driver genes such as *ARID1A*, *PIK3CA*, *KRAS*, and *PPP2R1A* in 26% of patients [32]. These findings suggest that reduced DNA repair capacity may predispose to genomic instability and progressive accumulation of somatic mutations [9,10]. The reduction in *GEN1* expression observed in our study indicates that a specific defect in the homologous recombination pathway may play a role in endometriosis pathophysiology.

Our findings have potential clinical and translational implications. The gold standard for endometriosis diagnosis remains invasive laparoscopic surgery, with an average diagnostic delay of 7–10 years [1,2]. Alterations in DNA repair gene expression present potential candidates for non-invasive diagnostic biomarker development. The Cochrane review by Nisenblat et al. emphasised that biomarker panels may enhance diagnostic performance, and *GEN1* expression could be evaluated as part of such a panel in future studies [33]. Furthermore, targeting DNA repair pathways offers new therapeutic opportunities. The successful use of PARP inhibitors in gynaecological cancers [31,34] suggests that similar approaches could be explored in endometriosis patients with DNA repair defects, although this remains speculative and would require extensive further investigation. A notable strength of this study is that endometriosis diagnosis was confirmed both laparoscopically and histopathologically, and controls were verified as disease-free during laparoscopic evaluation, ensuring robust group classification.

This study has several limitations. First, the sample size is relatively small (*n* = 3 per group), which limits statistical power; however, similar sample sizes are commonly employed in organoid studies [20,23,24]. This sample size is consistent with the exploratory nature of the study and comparable to other patient-derived endometriosis organoid studies that have employed limited numbers of independently derived organoid lines for functional analyses [24]. Additionally, detailed menstrual history parameters, including age of menarche and menstrual duration, were not systematically recorded; age of menarche was subject to recall bias and was not consistently available across participants, while menstrual duration demonstrated intra-individual variability that precluded reliable retrospective assessment. Furthermore, one control participant had hypertension and one had uterine myoma; although these conditions are not directly implicated in *GEN1* regulation, their potential influence on endometrial gene expression cannot be entirely excluded. Second, only advanced-stage (stage III–IV) endometriosis patients were included, and *GEN1* expression changes in early-stage patients could not be evaluated. Third, samples were collected only in the proliferative phase, and the effects of hormonal changes on *GEN1* expression could not be investigated. Fourth, endometrial organoids contain only epithelial cells and do not include stromal cells and immune cells [20]; future studies employing assembloid co-culture systems integrating stromal and immune components, or organ-on-chip platforms recapitulating the endometriotic microenvironment, would provide a more comprehensive understanding of *GEN1* function in disease pathophysiology [26]. Fifth, although γH2AX analysis was extended to include post-knockdown comparisons demonstrating increased DNA damage following *GEN1* silencing, the sample size remains small and further validation in larger cohorts is warranted. Finally, our study focused on a single DNA repair gene, and simultaneous evaluation of other homologous recombination pathway genes was not performed. Future studies should validate these findings in larger patient cohorts and across different menstrual cycle phases.

## 4. Materials and Methods

### 4.1. Ethical Approval and Tissue Collection

This study was approved by the Institutional Review Board of Biruni University Ethics Committee (Approval No: 2024-BİAEK/10-49; Date: 26 May 2025). Written informed consent was obtained from all participants prior to tissue sampling. The study was conducted in accordance with the principles of the Declaration of Helsinki (2013 revision). A total of six women were enrolled in this study between July and November 2025. The study population consisted of two groups: patients with laparoscopically confirmed endometriosis staged according to the revised American Society for Reproductive Medicine (rASRM) classification (*n* = 3; stage III, *n* = 1; stage IV, *n* = 2) and a control group without evidence of endometriosis confirmed by laparoscopic visualisation (*n* = 3). Inclusion criteria for both groups were as follows: women of reproductive age (18–45 years) with regular menstrual cycles (21–35 days). For the endometriosis group, a prior diagnosis confirmed by laparoscopic surgery and histopathological examination was required. For the control group, the absence of endometriosis was confirmed during laparoscopic surgery performed for benign indications. Exclusion criteria included hormonal therapy within the preceding three months (oral contraceptives, progestins, or GnRH analogues), suspected or confirmed endometrial hyperplasia or malignancy, active pelvic inflammatory disease or genital infection, pregnancy or lactation, history of autoimmune disease, diabetes mellitus or uncontrolled systemic disease, and use of medications that may affect the endometrium. Eutopic endometrial tissue was selected as the primary cell source for endometrial epithelial organoid (EEO) derivation.

Endometrial tissue samples were obtained by pipelle biopsy (Pipelle de Cornier, Laboratoire CCD, Paris, France) from the uterine cavity. Menstrual cycle phase was determined based on the calculated cycle day from the last menstrual period and confirmed by histological evaluation of endometrial dating by an experienced gynaecological pathologist. All samples were collected during the proliferative phase of the menstrual cycle (cycle days 6–14). Fresh tissue specimens were immediately placed in DMEM/F12 transport medium (Gibco, Thermo Fisher Scientific, Grand Island, NY, USA) supplemented with 1% penicillin–streptomycin and transported to the laboratory at 4 °C for processing within 2–4 h of collection.

### 4.2. Primary Cell Isolation and Organoid Culture

Endometrial tissue samples were processed using a combination of controlled mechanical dissociation and sequential enzymatic digestion. All reagents were thawed on ice prior to use, and Matrigel handling was performed at 4 °C to prevent premature polymerisation. Tissue samples were first minced into approximately 1–2 mm^3^ fragments using sterile surgical scissors in DMEM/F12 (Gibco) supplemented with 10% foetal bovine serum (FBS; Gibco) and 1% penicillin–streptomycin (Gibco). Tissue fragments were washed three times in warm Dulbecco’s phosphate-buffered saline (DPBS; Gibco) to remove blood and debris (300× *g*, 5 min, 4 °C).

Tissue fragments were subsequently transferred to a sterile enzymatic digestion solution containing 1 mg/mL collagenase type IV (Cat. No: C5138; Sigma-Aldrich, St. Louis, MO, USA) and 50 U/mL DNase I (Cat. No: 10104159001; Roche, Basel, Switzerland) in DMEM/F12. Digestion was performed at 37 °C in a shaking water bath for 60–90 min until partial dissociation was achieved. Following centrifugation (300× *g*, 5 min, 4 °C), residual extracellular matrix was further dissociated with 0.05% trypsin-EDTA (Gibco) for 10 min at 37 °C. Enzymatic activity was immediately neutralised using a solution containing 0.5 mg/mL soybean trypsin inhibitor (Sigma-Aldrich).

The resulting cell suspension was washed twice with complete DMEM/F12 medium and sequentially passed through 100 µm and 40 µm cell strainers (Corning, Corning, NY, USA). Epithelial glandular fragments retained on the 40 µm strainer were collected by inverting the strainer and backwashing with phenol red-free Advanced DMEM/F12 (Gibco). Cell number and viability were assessed using trypan blue exclusion with a haemocytometer. Isolated cells and glandular fragments were resuspended in ice-cold Matrigel Matrix Basement Membrane (Cat. No: 356234; Corning; lot number: 15525015; protein concentration: 10.6 mg/mL). Twenty-microlitre domes of the cell–Matrigel mixture were plated onto pre-warmed 48-well plates and allowed to polymerise at 37 °C for 15 min.

Following polymerisation, 250 µL of organoid expansion medium was added to each well. The expansion medium consisted of phenol red-free Advanced DMEM/F12 (Gibco) supplemented with 1% GlutaMAX (Gibco), 1% HEPES (Gibco), 1% penicillin–streptomycin, 1× B27 supplement (Gibco), 1× N2 supplement (Gibco), 1.25 mM N-acetylcysteine (Sigma-Aldrich), 50 ng/mL recombinant human EGF (PeproTech, Cranbury, NJ, USA), 100 ng/mL recombinant human FGF-10 (PeproTech), 100 ng/mL recombinant human Noggin (PeproTech), 500 ng/mL recombinant human R-spondin 1 (PeproTech), 500 nM A83-01 (Tocris, Bristol, UK), and 10 mM nicotinamide (Sigma-Aldrich). Y-27632 ROCK inhibitor (10 µM; Sigma-Aldrich) was included during the initial 48 h following plating and after each passage, then withdrawn from the culture medium for the remainder of the expansion period. Cultures were maintained under standard conditions (37 °C, 5% CO_2_, humidified atmosphere), and medium was changed every 48 h. Organoids were monitored daily for morphological development using an inverted phase-contrast microscope and passaged every 7–10 days by mechanical dissociation or using TrypLE Express (Gibco). The organoid isolation, culture, and expansion procedures described above were performed in accordance with the WERF EPHect-EM-Organoids standardisation guidelines for endometrial organoid research [26]. Organoid cultures were confirmed to be free of mycoplasma contamination by PCR-based testing prior to experimental use. All experiments were performed using EEOs between passages 2 and 5.

### 4.3. RNAi-Mediated Gene Silencing and Proliferation Assay

For *GEN1* gene silencing, EEOs were grown in culture for 7 days to allow the formation of compact, functional spheroids. Prior to transfection, EEOs were released from Matrigel by incubation with Cell Recovery Solution (Corning) for 30 min at 4 °C and dissociated into a single-cell suspension by gentle pipetting. Cells were then transfected with *GEN1* -specific small interfering RNA (siRNA) duplexes or non-targeting control siRNA at a final concentration of 20 nM using Lipofectamine RNAiMAX (Invitrogen, Thermo Fisher Scientific, Carlsbad, CA, USA) according to the manufacturer’s instructions.

The *GEN1*-targeting siRNA sequences were as follows: s132626, sense 5′-GGACUUAACAUUUAUGAGAtt-3′ and antisense 5′-UCUCAUAAAUGUUAAGUCCaa-3′; s132627, sense 5′-GGACAGUGCUAUGCUCGAAtt-3′ and antisense 5′-UUCGAGCAUAGCACUGUCCtt-3′ (Ambion, Thermo Fisher Scientific, Austin, TX, USA). A non-targeting siRNA duplex (Silencer Select Negative Control 1 siRNA, Cat. No: 4390843; Ambion) served as the negative control. These duplexes were selected based on pilot experiments demonstrating higher knockdown efficacy compared with other siRNA sequences.

For transfection, siRNA–Lipofectamine complexes were prepared in Opti-MEM (Gibco) and incubated at room temperature for 20 min. Cells were incubated with transfection complexes in suspension for 4 h at 37 °C, then re-embedded in fresh Matrigel and cultured in organoid expansion medium. In selected experiments, cells were co-transfected with 1 nM siGLO Red transfection indicator (Cat. No: D-001630-02; Dharmacon, Lafayette, CO, USA) to monitor transfection efficiency. Cells were harvested 48 h post-transfection, and knockdown efficiency was verified by RT-qPCR and Western blot analysis.

To assess cell proliferation, a BrdU incorporation assay was performed. siRNA-treated EEOs were labelled with 10 µM BrdU (Sigma-Aldrich) for 2 h at 48 h post-transfection. Following labelling, EEOs were released from Matrigel, dissociated into single-cell suspensions, and fixed with ice-cold 70% ethanol. DNA was denatured with 2 M HCl containing 0.5% Triton X-100 and neutralised with 0.1 M Na_2_B_4_O_7_ (pH 8.5). Cells were stained with FITC-conjugated anti-BrdU antibody (Cat. No: 11-5071-42; eBioscience, Thermo Fisher Scientific, San Diego, CA, USA) and propidium iodide (PI). BrdU incorporation was analysed by flow cytometry (BD FACSCanto II), and data were evaluated using FlowJo software (version 11; BD Biosciences). Each experiment was performed with at least three biological replicates.

### 4.4. Immunofluorescence and Confocal Microscopy

For immunofluorescence analysis, EEOs were first released from Matrigel by incubation with Cell Recovery Solution (Corning) at 4 °C for 30 min. Released EEOs were washed with PBS and fixed with 4% paraformaldehyde (PFA) for 30 min at room temperature. For whole-mount staining, fixed EEOs were washed three times with PBS and permeabilised overnight at 4 °C with PBS containing 0.5% Triton X-100 and 1% BSA. For primary cell cultures, cells were grown on glass coverslips and fixed with methanol for 10 min on ice. Fixed cells were permeabilised with 0.1% Triton X-100 and 0.5% NP-40 for 10 min on ice. To block non-specific binding, samples were incubated with PBS containing 5% normal goat serum and 1% BSA for 1 h at room temperature.

Samples were incubated with primary antibodies overnight at 4 °C. The primary antibodies used were as follows: rabbit anti-*GEN1* (1:400; Abcam, ab198989, Cambridge, UK) and mouse anti-E-cadherin (1:200; BD Biosciences, 610182, Franklin Lakes, NJ, USA). Following three washes with PBS, samples were incubated with secondary antibodies for 1 h at room temperature in the dark. The secondary antibodies were as follows: Alexa Fluor 488-conjugated goat anti-rabbit IgG (1:500; Invitrogen) and Alexa Fluor 594-conjugated goat anti-mouse IgG (1:500; Invitrogen). Following a final wash with PBS, samples were mounted with Vectashield antifade mounting medium containing DAPI (Vector Laboratories, Newark, CA, USA).

Confocal images were acquired using a Leica TCS SP5 microscope equipped with an HCX PL APO 63×/1.4 oil immersion objective. Dual-colour images were obtained using laser excitation at 488 nm and 561 nm for Alexa Fluor 488 and Alexa Fluor 594 dyes, respectively. Emission signals were collected through 500–550 nm and 590–650 nm band-pass filters. For DAPI, 405 nm laser excitation and 420–480 nm emission filter were used. Image acquisition and processing were performed using Leica LAS AF software (version 3.3; Leica Microsystems, Wetzlar, Germany). For fluorescence intensity quantification, ImageJ software (version 1.54q; NIH, Bethesda, MD, USA) was used, and at least 10 organoids or 50 cells per condition were analysed. For DNA damage assessment, γH2AX immunofluorescence was performed to evaluate baseline DNA double-strand break levels in control and endometriosis EEOs. EEOs were stained with rabbit anti-γH2AX (1:150; Thermo Scientific, MA5-33062, Waltham, MA, USA) following the same protocol described above. γH2AX immunofluorescence intensity was quantified in at least 50 nuclei per condition from three independent experiments using ImageJ software. In addition to baseline comparisons, γH2AX immunofluorescence was also assessed in EEOs at 48 h following transfection with *GEN1* -specific siRNA or non-targeting control siRNA to evaluate the effect of *GEN1* knockdown on DNA damage accumulation.

### 4.5. RNA Extraction and Quantitative Real-Time PCR

Total RNA was isolated from EEOs and primary cell cultures using TRIzol reagent (Invitrogen) according to the manufacturer’s instructions. Prior to RNA extraction, EEOs were released from Matrigel using Cell Recovery Solution (Corning) and washed with PBS. RNA concentration and purity were assessed using a NanoDrop ND-1000 spectrophotometer (Thermo Scientific), and samples with a 260/280 ratio between 1.8 and 2.0 were accepted for subsequent analyses. RNA integrity was verified by 1% agarose gel electrophoresis.

For complementary DNA (cDNA) synthesis, 1 µg of total RNA was reverse-transcribed using the High-Capacity cDNA Reverse Transcription Kit (Applied Biosystems) in a 20 µL reaction volume. The reaction mixture contained 50 U MultiScribe Reverse Transcriptase, 1× RT Buffer, 4 mM dNTP mix, 2.5 µM random hexamers, and RNase inhibitor. Reverse transcription was performed in a 2720 Thermal Cycler (Applied Biosystems) under the following conditions: 10 min at 25 °C, 120 min at 37 °C, and 5 min at 85 °C. The resulting cDNA samples were diluted 1:5 with nuclease-free water and stored at −20 °C.

Quantitative real-time PCR (RT-qPCR) was performed using the QuantStudio 3 Real-Time PCR System (Applied Biosystems). *GEN1* gene expression was analysed using a TaqMan Gene Expression Assay (*GEN1*, Hs00260513_m1; Applied Biosystems, Thermo Fisher Scientific, Foster City, CA, USA). S16 (Hs04332240_s1; Applied Biosystems) served as the internal control. Each 20 µL reaction mixture contained 10 µL TaqMan Universal PCR Master Mix II (No UNG), 1 µL TaqMan Gene Expression Assay (20×), 2 µL cDNA template, and 7 µL nuclease-free water. Thermal cycling conditions were as follows: 2 min at 50 °C, initial denaturation at 95 °C for 10 min, followed by 40 cycles of 15 s at 95 °C and 1 min at 60 °C. Each sample was run in technical duplicate, and nuclease-free water was included on each plate as a no-template control. Relative gene expression was calculated using the 2^−ΔΔCt^ method and normalised to the control group. Samples with cycle threshold (Ct) values above 35 were excluded from the analysis. Data were obtained from at least three independent biological replicates. To confirm hormonal receptor status of EEOs, oestrogen receptor alpha (*ESR1*, Hs00174860_m1; Applied Biosystems) and progesterone receptor (PGR, Hs01556702_m1; Applied Biosystems) expression was also assessed.

### 4.6. Western Blot Analysis

EEOs were released from Matrigel using Cell Recovery Solution (Corning) and lysed in RIPA buffer (Thermo Scientific) supplemented with protease and phosphatase inhibitor cocktail (Roche). Protein concentrations were determined using the BCA Protein Assay Kit (Pierce). Equal amounts of protein (20–30 µg) were separated by 10% SDS-PAGE and transferred onto PVDF membranes (Millipore, Burlington, MA, USA). Membranes were blocked with 5% non-fat milk in TBS-T for 1 h at room temperature and incubated overnight at 4 °C with primary antibodies: rabbit anti-*GEN1* (1:1000; Abcam, ab198989, London, UK) and mouse anti-β-actin (1:5000; Sigma-Aldrich, A5441). After washing with TBS-T, membranes were incubated with HRP-conjugated secondary antibodies for 1 h at room temperature: goat anti-rabbit IgG-HRP (1:5000; Cell Signaling) and goat anti-mouse IgG-HRP (1:5000; Cell Signaling). Protein bands were visualised using ECL substrate (Bio-Rad, Hercules, CA, USA) and quantified by densitometry using ImageJ software (NIH). β-Actin served as the loading control.

### 4.7. Statistical Analysis

All statistical analyses were performed using IBM SPSS Statistics version 26.0 (IBM Corp., Armonk, NY, USA) and GraphPad Prism version 9.0 (GraphPad Software). Due to the small sample size (*n* = 3 per group), the non-parametric Mann–Whitney U test was used for between-group comparisons. Continuous variables were expressed as mean ± standard deviation (SD). With *n* = 3 per group, the exact Mann–Whitney U test yields a minimum two-tailed *p*-value of 0.05 only when complete separation between groups is observed (U = 0). Therefore, results were interpreted considering both statistical significance and the magnitude of observed effects. For RNAi-mediated knockdown experiments, silencing efficiency was reported descriptively as percentage reduction ± SD, as paired within-subject comparisons with *n* = 3 do not provide sufficient statistical power for formal hypothesis testing.

For gene expression analyses, control group values were normalised to 1.00, and endometriosis group values were calculated relative to this reference. RT-qPCR data were analysed using th 2^−ΔΔCt^ method with S16 as the reference gene. Western blot band intensities were measured densitometrically using ImageJ software (National Institutes of Health) and normalised to β-actin. EEO formation efficiency was calculated as the ratio of formed EEOs to total seeded cells and expressed as a percentage. EEO diameters were determined by measuring at least 50 EEOs per group using ImageJ. For proliferation analyses, BrdU incorporation was quantified by flow cytometry and expressed as a percentage of BrdU-positive cells. *GEN1* knockdown efficiency was determined as the percentage reduction in mRNA and protein levels compared with the control siRNA group.

All experiments were performed with at least three independent biological replicates, and technical duplicates were included for each experiment. Statistical significance was defined as *p* ≤ 0.05. Given the small sample size, exact *p*-values are reported throughout. With *n* = 3 per group, the minimum achievable *p*-value for the Mann–Whitney U test is 0.05, corresponding to complete rank separation between groups.

### 4.8. Use of Artificial Intelligence Tools

During the preparation of this manuscript, the authors used ChatGPT Plus, a large language model–based artificial intelligence tool, solely for language editing and refinement, and manuscript for-matting. All AI-assisted outputs were reviewed, verified, and, where necessary, revised by the authors. Full responsibility for the scientific content, interpretations, and conclusions of this manuscript rests with the authors.

## 5. Conclusions

To our knowledge, this study provides the first organoid-based evidence that *GEN1*, a critical Holliday junction resolvase, is downregulated in endometriosis and functionally associated with reduced cell proliferation in vitro. Endometriosis-derived EEOs exhibited elevated baseline DNA damage and heightened sensitivity to *GEN1* knockdown compared with controls, suggesting an underlying genomic fragility that may contribute to disease pathogenesis. The convergence of reduced *GEN1* expression, elevated baseline DNA damage, and heightened sensitivity to further *GEN1* depletion supports internal consistency of the observed phenotype. These findings suggest a potential contribution of homologous recombination dysregulation to endometriosis pathogenesis and support further investigation of DNA repair mechanisms in endometriosis. Patient-derived EEOs represent a powerful translational platform for elucidating disease mechanisms and developing personalised treatment strategies.

## Figures and Tables

**Table 1:** Clinical and demographic characteristics of study participants.

Characteristic	Control (*n* = 3)	Endometriosis (*n* = 3)	*p*-Value
Age (years)	30.8 ± 3.6 (27.2–34.4)	32.4 ± 4.2 (28.2–36.6)	NS
BMI (kg/m^2^)	23.2 ± 2.1 (21.1–25.3)	24.1 ± 2.5 (21.6–26.6)	NS
Menstrual cycle day	9.3 ± 1.5 (8–11)	9.7 ± 1.2 (9–11)	NS
Cycle phase	Proliferative	Proliferative	–
Gravidity	1.7 ± 0.6 (1–2)	1.0 ± 1.0 (0–2)	NS
Parity	1.0 ± 0.0 (1–1)	0.3 ± 0.6 (0–1)	NS
Previous pelvic surgery, *n* (%)	3 (100%)	3 (100%)	–
Primary infertility, *n* (%)	1 (33.3%)	2 (66.7%)	–
**Indication for surgery**			
Endometriosis	–	3 (100%)	–
Benign ovarian cyst	2 (66.7%)	–	–
Tubal ligation	1 (33.3%)	–	–
**rASRM stage, *n* (%)**			
Stage III	–	1 (33.3%)	–
Stage IV	–	2 (66.7%)	–
**Comorbidities, *n* (%)**			
Hypertension	1 (33.3%)	0 (0%)	–
Uterine myoma	1 (33.3%)	0 (0%)	–

Data are presented as mean ± SD (min–max) or *n* (%). NS, not significant (*p* > 0.05, exact Mann–Whitney U test). –, not applicable. rASRM, revised American Society for Reproductive Medicine.

## Data Availability

The data presented in this study are available on request from the corresponding author. The data are not publicly available due to patient confidentiality and ethical restrictions.
